# Evaluation of Quality of Life of the Elderly Population Covered by Healthcare Centers of Marivan and the Influencing Demographic and Background Factors in 2010

**DOI:** 10.5812/ircmj.1834

**Published:** 2012-11-15

**Authors:** Fereshteh Farzianpour, Mohammad Arab, Seyyed Mustafa Hosseini, Bakhtiar Pirozi, Shadi Hosseini

**Affiliations:** 1 Department of Health Management and Economic, School of Public Health, Tehran University of Medical Sciences, Tehran, Iran; 2 Department of Epidemiology and Statistic, School of Public Health, Tehran University of Medical Sciences, Tehran, Iran; 3 Department of Health Management, Zabol University, Zabol, Iran

**Keywords:** Quality of Life, Healthcare

Dear Editor,

World Health Organization reported there are more than 600 million elderly individuals in the world (1); a figure which will double by 2025 and will reach 2 billion people by 2050 (2, 3).The immense changes in cultural, socioeconomic and demographic aspects of Iranian society during the last few decades have given rise to an increase in the elderly population (2).

A review on the age structure of Iranian population during the last half century reveals the increase in number and percentage of elderly individuals (2): the elderly population of Iran has almost doubled from 3.9% in 1956 to 7.3% (5121043 individuals) in 2006 (2). The rapid, unprecedented growth of elderly population in developing countries, which are still in fight with poverty and contagious diseases, is posing an obstacle for development, thus necessitating urgent measures and approaches for remedy (3). In many of these countries, poverty, deficits in social security programs, continuous urbanization and ever-growing recruitment of women as workforce all compromise the traditional forms of caring for the elderly people (3). It is said that an aging population is the consequence of development; however, if we are unprepared for this phenomenon in a developed world, it will lead to many complications (3-5).Therefore, the international community is paying greater attention to encountering the adverse outcomes of this phenomenon through adoption of appropriate policies in order to enhance the physical, mental and social status of the elderly population i.e. improve their quality of life (QOL) (5-7).

This study evaluated QOL of the elderly population covered by healthcare centers in Marivan, Iran. The study population consisted of all the elderly covered by 5 healthcare centers and 2 healthcare bases in Marivan, summing up to 2,433 individuals. A total of 400 these elderly were studied. A simple stratified random sampling proportional to the size of healthcare centers was performed. Data were collected using a standardized QOL questionnaire (Short Form Health Survey (SF-36) – Iranian Version) (8) and questions on demographic and background factors which were completed through face-to-face interviews. The SF-36 questionnaire has been translated and used in over 50 countries all over the world including Iran. The questionnaire consists of two major scales: the physical component and the mental component. The SF-36 also consisting 8 subscales (domains): physical functioning, role physical, bodily pain, general health, vitality, social functioning, and role emotional and mental health. Each of these 8 dimensions may be scored from 0 through 100. Scoring is performed according to criteria of SF-36 standards, with higher scores indicating better function (8). Data were entered and analyzed in SPSS (16.0), using non-parametric Mann-Whitney U test and Kruskal-Wallis test. Then multiple regressions were performed.

240 (60%) of the elderly were men and 160 (40%) were women. 76.3% of the elderly were 60-69 years old and 87.2% of them illiterate. 18% of the elderly stated they have financial problems however 19.5% did not express any financial problems. Most of them (72.7%) were living with their spouse and/ children and 74.8% were married. The mean in eight domains of QOL were as follows: Physical Functioning: 53.2; Role-Physical: 31.6; Bodily Pain: 51; General Health: 47.1; Vitality: 52.4; Social Functioning: 60; Role Emotional: 40.9; Mental Health: 61.4. In general, the physical and mental components scored 45.7±21.8 and 53.7±23.7, respectively, yielding an overall score of 49.7±20.6 for the couplet indices of quality of life ( Table 1 ).

**Table 1:** Mean score of QOL of the elderly population covered by healthcare centers in Marivan, Iran - 2010

Indices of Quality of Life (QOL)	Mean ± SD
**Physical Functioning**	53.2 ± 29.1
**Role Physical**	31.6 ± 38.1
**Bodily Pain**	51 ± 23.5
**General Health**	47.1 ± 19.6
**Vitality**	52.4 ± 20.3
**Social Functioning**	60 ± 30.3
**Role Emotional**	40.9 ± 42.4
**Mental Health**	61.4 ± 19.7
**Physical Component**	45.7 ± 21.8
**Mental Component**	53.7 ± 23.7
**Overall Quality of Life (QOL)**	49.7 ± 20.6

The impacts of gender, age, education, financial and marital status, status of living on the mean scores of 8 subscales of QOL were studied using the Mann-Whitney U test and Kruskal-Wallis test. The mean of all 8 subscales of QOL were significantly lower for women compared to men (P < 0.001; Mann-Whitney U test) except in the case of role physical (P = 0.62). Also, the mean of all 8 subscales of QOL decreased significantly (P < 0.001) with increase in age, except for the case of vitality (P = 0.28). The analysis of the relationship between financial status with subscales of QOL showed that for all subscale the corresponding mean scores are significantly higher (P ≤ 0.032) for those who financially doing better. Moreover, the mean of 8 QOL subscale scores are significantly (P < 0.005) higher for the diploma and higher educated elderly. The mean of all subscales are also significantly (P < 0.003) higher for the elderly living with their spouse and or children and are worse for whom were widowed.

Multiple regression for all 8 subscales of the QOL were carried out to identify which of the gender, age, education, financial and marital status, status of living were the most important independent factors after adjusting for the effect of possible confounders we found that education, financial and marital status were the most common important independent factors on each of the 8 subscales of QOL. Our findings support previously described beneficial effects of the counseling model on the elderly QOL in the cities of Masjed solaiman (5), Zahedan (9) and Shahinshar (10), Bandar Abbas (11), Iran. The mean QOL scores measured in the elderly population, in other countries were much higher than the results obtained in Iran (12). The QOL subscales were influenced by different factors including age, gender, financial status and more importantly by education, financial and marital status as other studies showed (5, 13, 14). Therefore, it is important to inform the elderly population of the behavioral modifications benefits of the QOL.
